# Retraction Note to: Activation of LncRNA TINCR by H3K27 acetylation promotes Trastuzumab resistance and epithelial-mesenchymal transition by targeting MicroRNA-125b in breast Cancer

**DOI:** 10.1186/s12943-022-01609-6

**Published:** 2022-06-29

**Authors:** Huaying Dong, Jianguo Hu, Kejian Zou, Mulin Ye, Yuanwen Chen, Chengyi Wu, Xin Chen, Mingli Han

**Affiliations:** 1 Department of General Surgery, Hainan General Hospital, Hainan Medical University, No.19 Xiu Hua Road, Xiuying District, Haikou City, 570311 Hainan Province China; 2 Department of Obstetrics and Gynecology, The Second Affiliated Hospital, Chongqing Medical University, Chongqing, 400010 China; 3 Department of General Surgery, Chongqing Renji Hospital, University of Chinese Academy of Science, Chongqing, 400062 China; 4 Department of General Surgery, The Frist Affiliated Hospital, Chongqing Medical University, Chongqing, 400016 China; 5 Department of Breast Surgery, The First Affiliated Hospital of Zhengzhou University, Zhengzhou, 450052 China

**Retraction note to: Mol Cancer 18, 3 (2019)**

**https://doi.org/10.1186/s12943-018-0931-9**

The Editor-in-Chief has retracted this article. After publication, concerns were raised regarding image overlap between Figs. 1f and 3d, which the authors address by a Correction [1]. However, further concerns have been raised about potential image overlap with two other articles [2, 3]. Specifically, in Fig. 1g, one of the subpanels appears to be highly similar to an image in Fig. 5a in ([2], now retracted). In Fig. 3e, the top three images appear highly similar to the bottom three images (rotated) in Fig. 2f in [3].

The authors have provided the original data to address the concerns. However, the original data contains overlapping cell images and highly similar backgrounds in the western blot gel photographs. Additionally, the source, number and sex of animals are different between the Ethics approval document and the Methods section of the article.

The Editor-in-Chief therefore no longer has confidence in the presented data and the ethical standards of the study.

Huaying Dong has stated on behalf of all co-authors that they agree to this retraction.
